# V(D)J recombination process and the Pre-B to immature B-cells transition are altered in *Fanca*^*−/−*^ mice

**DOI:** 10.1038/srep36906

**Published:** 2016-11-24

**Authors:** Thuy Vy Nguyen, Patrycja Pawlikowska, Virginie Firlej, Filippo Rosselli, Saïd Aoufouchi

**Affiliations:** 1 CNRS UMR 8200 – Gustave Roussy, Villejuif, France; 2 Université Paris Saclay, Orsay, France; 3 Equipe Labellisée Ligue Contre le Cancer, CNRS UMR8200, Villejuif, France; 4 Université Paris Est, Créteil, Val de Marne, France; 5 Université Paris Descartes, Paris, France

## Abstract

B-lymphocytes in the bone marrow (BM) must generate a functional B-cell receptor and overcome the negative selection induced by reactivity with autoantigens. Two rounds of DNA recombination are required for the production of functional immunoglobulin heavy (Ig-HCs) and light (LCs) chains necessary for the continuation of B-lymphocyte development in the BM. Both rounds depend on the joint action of recombination activating gene-1 (RAG-1) and RAG-2 endonucleases with the DNA non-homologous end-joining pathway. Loss of the *FANC* gene leads to the chromosome breakage and cancer predisposition syndrome Fanconi anemia. Because the FANC proteins are involved in certain aspects of the recombination process, we sought to determine the impact of the FANC pathway on the Ig diversification process using *Fanca*^−/−^ mice. In this work we demonstrated that *Fanca*^−/−^ animals have a mild B-cell differentiation defect characterized by a specific alteration of the IgM^−^ to IgM^+^ transition of the B220^low^ B-cell population. Pre-B cells from *Fanca*^−/−^ mice show evidence of impaired kLC rearrangement at the level of the Vk-Jk junction. Furthermore, *Fanca*^−/−^ mice showed a skewed Vκ gene usage during formation of the LCs Vk-Jk junctions. Therefore, the Fanca protein appears as a yet unidentified factor involved in the primary diversification of Ig.

To cope with the enormous variety of pathogens and to recognize non-self molecules, B-cells have evolved controlled genetic processes at their immunoglobulin (Ig) loci known as Ig diversification. Primary diversification occurs during early B-cell development in the bone marrow (BM) via the assembly of a complete IgM antigen receptor exposed on the B-cell surface (BCR) by a site-specific recombination process called V(D)J recombination. Mature B-cells that express a functional IgM migrate from the BM to the periphery, where antigen-dependent secondary diversification occurs following two activation-induced cytidine deaminase-dependent processes known as somatic hypermutation and class switch recombination (CSR)^1^.

To produce the Ig heavy chain (HC), V(D)J recombination starts in the BM at the pro-B cell stage by the D-to-J_H_ rearrangement followed by the V_H_-to-DJ_H_ rearrangement. Productive HC rearrangement leads to IgM-HC expression. After the assembly of the IgM-HC with a surrogate light chain (LC) and CD79a and b proteins, the IgM-HC is exposed on the cell surface as the precursor-B cell receptor (pre-BCR). Signals from the pre-BCR orchestrate the proliferation and subsequent developmental transition to the small pre-B-cell stage, where Igκ or Igλ LC VJ recombination is initiated^2^,^3^. Successful pairing of a productive LC with an IgM-HC results in the expression of a BCR at the cell surface and progression to immature B cells, which are checked for autoreactivity before leaving the BM^4^.

V(D)J recombination depends on the action of the lymphoid-specific RAG-1 and RAG-2 endonucleases that initiate DNA cleavage at defined recombination signal sequences (RSS) that flank the V, D, and J gene segments. The RAG complex mediates the formation of two hairpinned extremities, called coding ends (CEs), cutting-off a DNA segment creating an one-ended blunted DSB at each extremity, at the signal end^5^,^6^. The signal ends of a DNA fragment are sealed by non-homologous end joining (NHEJ), and the formed circle is displaced. The two hairpins are opened by the endonuclease Artemis in association with activated DNA-PKcs^7^ and joined by the complex formed by XRCC4, LIG4 and XLF/Cernunnos. Due to the intrinsic error prone property of NHEJ, the obtained coding joints frequently lose and/or gain nucleotides^8^. While the loss of nucleotides is a consequence of the 5′ and 3′ overhang modification by Artemis^7^, a nucleotide gain results from the template-independent activity of the terminal deoxynucleotidyl transferase (TdT) DNA polymerase (N nucleotide additions) or from the activities of DNA polymerases operating on the hairpin that has been opened asymmetrically (P nucleotide additions)^9^,^10^,^11^,^12^,^13^. The junction of regions from the V, D and J segments encodes the CDR3, the major determinant of the antigen binding site specificity. V(D)J is a highly regulated process that ensures the development of a normal immune system and prevents potential oncogenic events such as translocations, during the sealing step of the CE.

Fanconi anemia (FA) is a rare inherited disorder characterized by chromosome breakage, cancer predisposition and BM failure^14^,^15^. The syndrome is genetically heterogeneous, and twenty *FANC* genes (named *A* to *U*) have been identified to date^14^,^16^,^17^. The major and most robust role of the FANC pathway is its involvement in the DNA damage response. Following DNA damage or replicative stress, eight upstream FANC proteins (FANCA, FANCB, FANCC, FANCE, FANCF, FANCG, FANCL, and FANCM) assemble into the “FANCcore complex”, which, together with FANCT/UBE2T, is necessary for the monoubiquitination and nuclear foci formation of both FANCD2 and FANCI. The monoubiquitinated FANCD2/FANCI heterodimer functionally and/or biochemically interacts with the downstream FANC proteins FANCD1/BRCA2, FANCN/PALB2, FANCJ/BRIP1, FANCO/RAD51C, FANCP/SLX4, FANCQ/XPF, FANCR/RAD51, FANCS/BRCA1 and FANCU/XRCC2 to eliminate DNA lesions and/or to rescue stalled replication forks^14^,^18^,^19^. Accumulating evidence indicates that FA proteins function to coordinate DNA double-strand breakage repair activity by regulating homologous recombination and/or NHEJ^20^,^21^,^22^. Studies using model organisms, cells and cellular extracts have revealed that altered DNA end joining activities occur in the absence of a FANC protein^21^,^23^,^24^. Recently, we have shown that Fanca is required during CSR to stabilize duplexes between pairs of short microhomology regions located at DNA ends^25^. Because DNA end-joining activities are a prerequisite for primary immunoglobulin diversification, we reasoned that the FANC pathway could be involved in these mechanisms. To address this question, we analysed B-cell development and V(D)J recombination in *Fanca*^−/−^ mouse-derived B-cells.

## Results

### Impaired IgM^−^ to IgM^+^ transition in bone marrow lymphoid cells isolated from *Fanca* ^−/−^ mice

**Figure 1:**
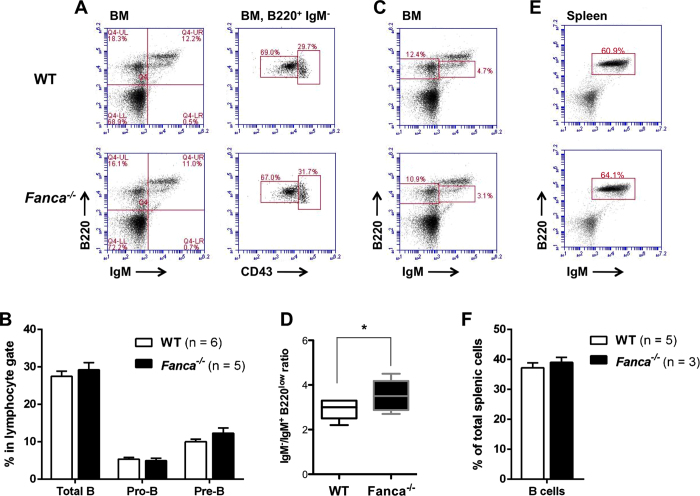
*Fanca*^−/−^ mice show moderate impairment of the IgM^−^ to IgM^+^ transition in the BM. (**A**) A representative flow cytometry plot used to identify and estimate the frequency of B220^+^ (total B), B220^+^ IgM^−^ CD43^low^ (pre B) and B220^+^ IgM^−^ CD43^high^ (pro **B**) cells isolated from WT and *Fanca*^−/−^ mouse BM. (**B**) Quantification of the data in A (mean ± SEM; n = 5 par genotype). (**C**) A representative flow cytometry plot used to identify and estimate the frequency of IgM^−^ B220^low^ and IgM^+^ B220^low^ populations of BM cells from WT and *Fanca*^−/−^ mice. (**D**) The ratio of IgM^−^ B220^low^ cells and IgM^+^ B220^low^ immature B cells identified as shown in C. (n = 8 for *Fanca*^−/−^ mice; n = 7 for WT mice *p < 0.05). (**E**) Flow cytometry representation of B220^+^ IgM^+^ cells derived from WT and *Fanca*^−/−^ mouse spleen. (**F**) Average percentage of B cells (B220^+^ IgM^−^) in the spleen of WT and *Fanca*^−/−^ mice (mean ± SEM).

To determine whether Fanca is involved in the mechanism of V(D)J recombination in B cells, we first analysed lymphoid tissue development in the BM and spleen of 8-week old *Fanca*^−/−^ and WT mice. Flow cytometry analysis of the BM showed no quantitative difference in the proportion of total B cells (B220^+^), pro-B cells (B220^+^ IgM^−^ CD43^high^), and pre-B cells (B220^+^ IgM^−^ CD43^low^) between *Fanca*^−/−^ and WT mice (Fig. 1A,B).

However, we observed a significantly increased ratio of IgM^−^ (IgM^−^ B220^low^) to immature B cells (IgM^+^ B220^low^) in *Fanca*^−/−^ mice (3.5 in *Fanca*^−/−^ mice vs. 2.9 in WT, Fig. 1C,D), suggesting that the transition from IgM^−^ to immature B cells was somewhat defective. Indeed, the *Fanca*^−/−^ mice had a mild accumulation (15.9% vs 15.2% of total lymphocytes) in BM B cells at the IgM^−^ stage and consequently showed a reduction in the proportion of immature B cells (Table S1). However, the proportion of mature, recirculating B cells (IgM^+^ B220^high^) was unchanged in the absence of Fanca (Table S1), indicating that subsequent maturation steps in the IgM^+^ compartment are unaffected.

Consistent with the absence of differences in mature B cells compartment in the BM, an analysis of total B cells in the spleen showed that the percentage of B cells was also similar between WT and *Fanca*^−/−^ mice (Fig. 1E,F).

### Impaired P-addition process during HC rearrangement in *Fanca* ^−/−^ mice

**Figure 2:**
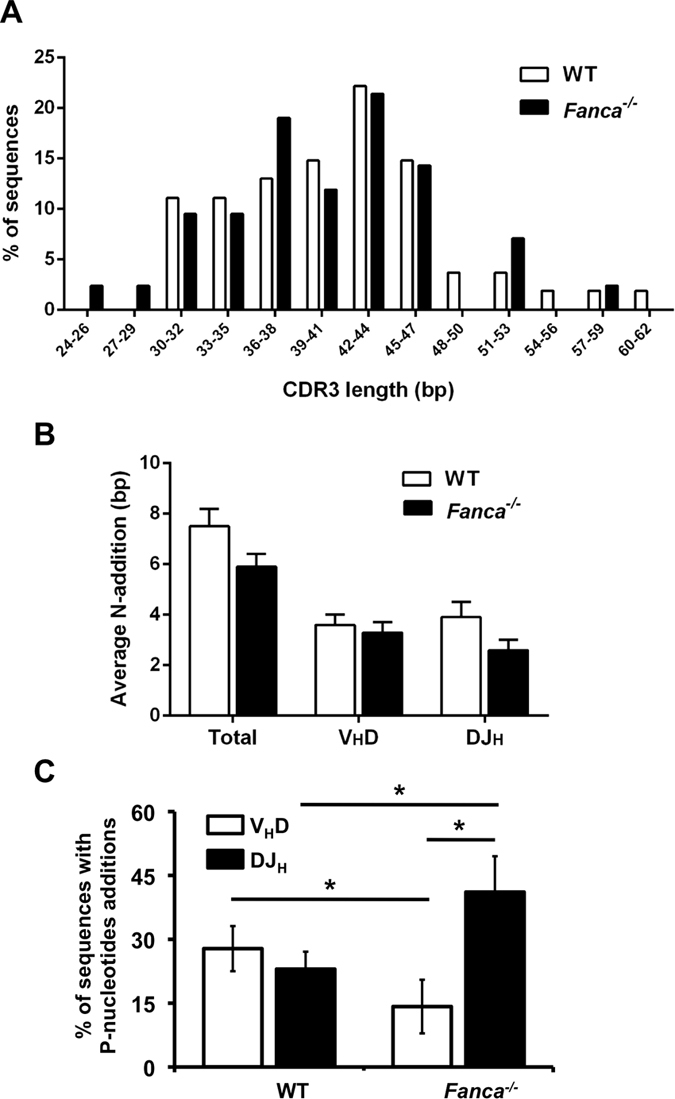
Normal heavy chain rearrangement in *Fanca*^−/−^ mice. (**A**) Distribution of CDR3 lengths of V_H_DJ_H4_ rearrangements amplified from genomic DNA that was isolated from BM IgM^−^ B cells of *Fanca*^−/−^ and WT mice. The average values are listed in Table 1. (**B**) Average numbers of added N-nucleotides per sequence in V_H_DJ_H4_ rearrangements of *Fanca*^−/−^ and WT mice are plotted either for V_H_ to DJ_H_ and D to J_H_ junctions (“Total”) or for V_H_ to DJ_H_ (“V_H_D”) alone or D to J_H_ (“DJ_H_”) junctions alone. The results are displayed as the mean ± SEM. (**C**) The proportion of P-nucleotide additions at either the V_H_ to DJ_H4_ (“V_H_D”) or the D to J_H4_ (“DJ_H_”) junctions. The data for HC rearrangements are from three independent pools of three mice per genotype (the numbers of sequences analysed are indicated in Table 1).

To obtain a better characterization of the transition from the pro-B (the phase where HC rearrangement occurs) to the pre-B stage in Fanca deficient cells, we addressed V(D)J rearrangement efficiency by a PCR-based assay using V_H_ and J_H4_ consensus primers to amplify HC-rearranged junctions in DNA isolated from *Fanca*^*+/+*^ and *Fanca*^−/−^ BM B220^+^ IgM^−^ cells. The rearranged junctions were analysed for length, size range and junctional diversity of CDR3. Our data showed that the average length, size range and length distribution of CDR3 of rearranged VDJ_H4_ genes as well as the proportion of in-frame (vs. out-of–frame sequences were similar between *Fanca*^−/−^ and WT mice (Table 1 and Fig. 2A). A determination of the length of each V, D, and J segment contributing to the CDR3 region showed no considerable difference between WT and the *Fanca*^−/−^ mice (Table S2). Additionally, we found that the average number of N-additions, whether estimated for both the V_H_-D and D-J_H_ junctions (total) or for either the V_H_-D or the D-J_H_ junction alone appeared to be similar between the two groups of mice (Fig. 2B). On the contrary, P-additions at the V_H_-D or D-J_H_ junctions differ significantly between *Fanca*^−/−^ and WT mice (Fig. 2C). Surprisingly, whereas the ratio of P-additions at D-J_H_ vs V_H_-D junctions was similar in WT cells (0.86+/−0.2), a significant disequilibrium was observed in Fanca^−/−^ cells (ratio of 3.11+/−0.8). In other words, in *Fanca*^−/−^ B cells, we observed 3 times more sequences with P-additions at D-J_H_ junctions than at V_H_-D.

Consequently, even if our data demonstrated that *Fanca*^−/−^ mice are competent in the transition from pro-B to pre-B stage, they uncover a still undetermined role for Fanca during the early step of V(D)J rearrangement of the HCs.

### *Fanca* ^−/−^ mice accumulate in-frame Vκ-Jκ1 junctions in BM IgM^−^ B cells

**Figure 3:**
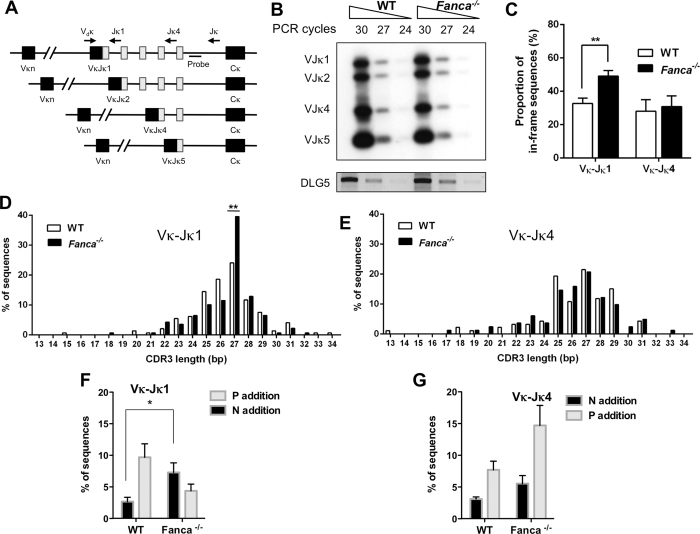
*Fanca*^−/−^ mice accumulate in-frame Vκ-Jκ1 junctions in BM IgM^−^ B cells. (**A**) Schematic diagrams (not to scale) of the PCR assays used to determine LC rearrangement. The top map shows the positions of a degenerated V_d_κ gene 5′ primer and Jκ1, Jκ4 and Jκ 3′ primers along with the position of a probe used in Southern blotting. Below are shown the four possible rearranged products resulting from Vκ joining to the different Jκ gene segments. (**B**) Semi-quantitative PCR at different cycles and a Southern blot analysis of the rearrangements of Vκ gene segments to Jκ1 to Jκ5 gene segments in IgM^−^ B cells sorted from the BM of *Fanca*^−/−^ and WT mice. The DNA input was normalized to DLG5 PCR products (below). Original images were reported in Supplemental Figure S4. (**C**) Proportions of in-frame Vκ-Jκ1 and Vκ-Jκ4 rearrangements amplified from genomic DNA isolated from BM IgM^−^ B cells of *Fanca*^−/−^ and WT mice. The data are displayed as the mean ± SEM of 4 mice per genotype from 4 independent experiments for Vκ-Jκ1 rearrangements (**p < 0.01 with a 2-tailed Student’s paired t-test) and 3 mice per genotype from 3 independent experiments for Vκ-Jκ4 rearrangements. (**D**,**E**) Distribution of CDR3 lengths of Vκ-Jκ1 and Vκ-Jκ4 rearrangements from BM IgM^−^ cells of *Fanca*^−/−^ and WT mice, respectively (**p < 0.01 with Fisher’s exact test). The average values and numbers of the sequences analysed are listed in Table 1. (**F**,**G**) Proportions of P- and N-nucleotide additions in the Vκ-Jκ1 and Vκ-Jκ4 rearrangements, respectively, from BM IgM^−^ cells of *Fanca*^−/−^ and WT mice. The data are displayed as the mean ± SEM of 4 mice per genotype from 4 independent experiments for Vκ-Jκ1 rearrangements and 3 mice per genotype from 3 independent experiments for Vκ-Jκ4 rearrangements.

Having shown that *Fanca*^−/−^ mice harbour a defect in the pre-B to immature B-cell transition (Fig. 1C,D), a step that requires a successful LC rearrangement, we hypothesized that Fanca could be specifically involved in the regulation of VJ recombination at the LC locus. To test this hypothesis, we first assessed the Igκ rearrangement efficiency in WT and *Fanca*^−/−^ BM IgM^−^ B cells using PCR. A degenerate Vκ primer (V_d_κ VκD) that binds to ∼90% of Vκ gene segments was used together with a primer downstream of Jκ5 (Jκ; Fig. 3A). A genomic sequence within the murine DLG5 gene was used to normalize the DNA input. The intensities of PCR bands for Vκ-Jκ rearrangements were comparable in IgM^−^ B cells from WT and *Fanca*^−/−^ mice (Fig. 3B) indicating that a Fanca deficiency does not detectably affect the Igκ LC recombination step. We next sequenced and analysed the Vκ-Jκ1 (proximal) and Vκ-Jκ4 (distal) rearranged junctions from BM-sorted B220^+^ IgM^−^ cells that had not yet produced a functional B cell receptor. The Vκ-Jκ1 and Vκ-Jκ4 junctions represented the primary and secondary rearrangements during Igκ LC recombination, respectively. A V_d_κ primer and a downstream primer Jκ1 or Jκ4 were used to amplify the Vκ-Jκ1 or Vκ-Jκ4 junctions, respectively (Fig. 3A). Rearranged junctions were analysed for length, size range and junction diversity of CDR3. Our results showed that the average length and size range of CDR3 of both the Vκ-Jκ1 and the Vκ-Jκ4 rearrangements in BM B220^+^ IgM^−^ cells were similar between *Fanca*^−/−^ and WT mice (Table 1). Nevertheless for the Vκ-Jκ1 rearrangement, we observed that the ratio of in-frame vs. out-of-frame sequences was 0.47 for WT mice and 0.92 for *Fanca*^−/−^ mice. In other words, 32% of the analysed CDR3 sequences from WT animals in IgM^−^ B220^+^ B-cells are in-frame compared with 48% in *Fanca*^−/−^ mice, whereas no difference was noticed for the Vκ-Jκ4 rearrangements (Fig. 3C). The increased proportion of in-frame Vκ-Jκ1 sequences in *Fanca*^−/−^ mice could simply be a reflection of the accumulation of the IgM^−^ B-cells that we previously observed. However, only the Vκ-Jκ1 rearrangements appear unbalanced, suggesting a specific role of Fanca in their joining. A noticeable effect of a Fanca deficiency is evident on the histogram in Fig. 3D, which shows the observed CDR3 sizes. The canonical length of 27 nucleotides was observed in 40% of the Vκ-Jκ1 CDR3 from *Fanca*^−/−^ mice compared with 24% retrieved from WT. On the other hand, the distribution of CDR3 lengths in Vκ-Jκ4 rearrangements was similar between two groups of mice (Fig. 3E). Further analysis of the Igκ rearranged junctions, indicated that even if the global frequency of sequences showing nucleotides addition was similar (less than 15%) for the Vκ-Jκ1 rearrangement between WT and *Fanca*^−/−^ mice, the average of N addition was significantly higher in *Fanca*^−/−^ mice and the proportion of P-additions vs. N-additions was clearly reversed (Fig. 3F). Again as a supplementary clue of specificity, the relative proportion of P-additions vs. N-additions in the Vκ-Jκ4 junctions was similar between the WT and *Fanca*^−/−^ mice (Fig. 3G).

**Figure 4:**
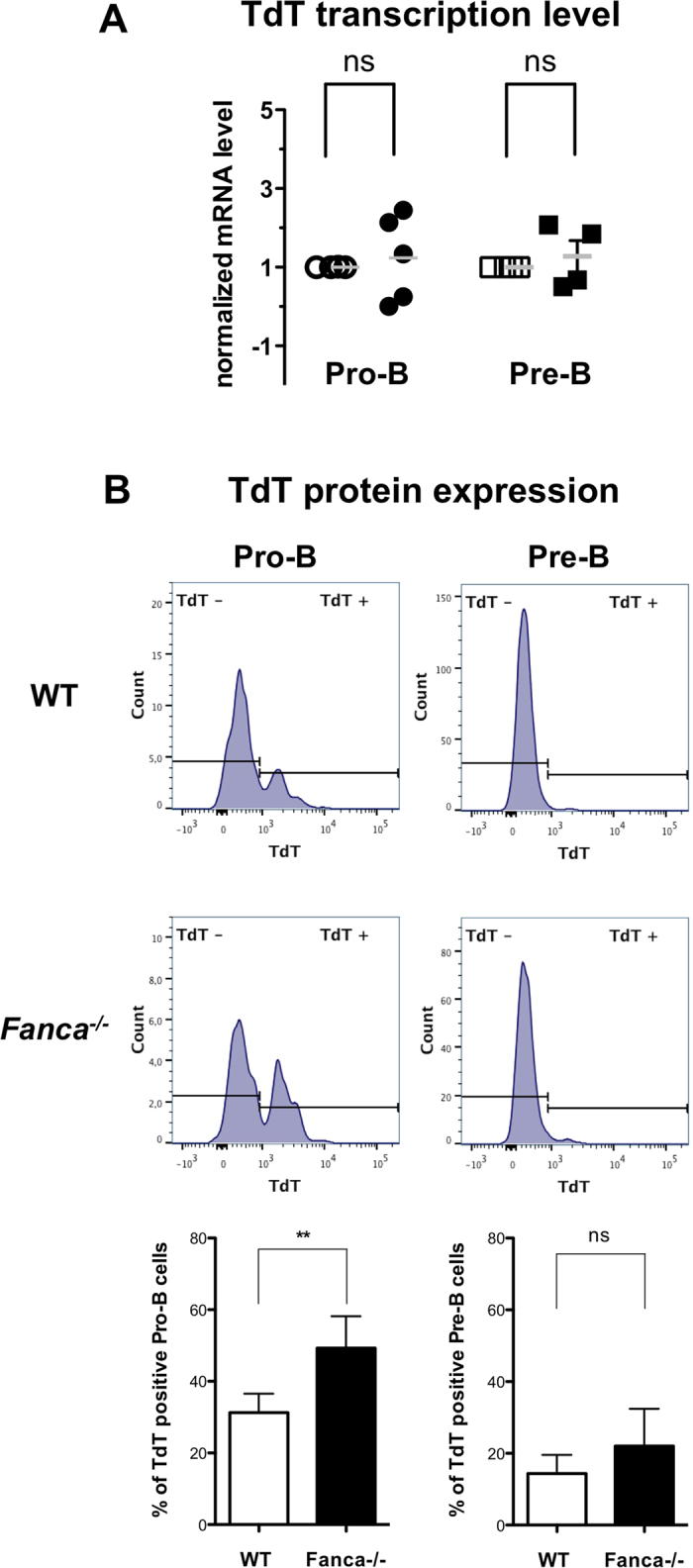
Fanca deficiency increases TdT protein expression in pro-B and pre-B cells. (**A**) Quantitative reverse transcriptase-polymerase chain reaction analysis of TdT transcript expression in sorted pro-B and pre-B *Fanca*^−/−^ cells. Open symbols represent WT mice and filled symbols represent Fanca^−/−^ mice. The results were calculated relative to the WT and normalized against the level of actin (mean ± SEM; n = 4 for pre-B and n = 5 for pro-B cells). (**B**) TdT protein expression estimated by flow cytometry (the gating strategy is shown in Fig. S2) in pre-B (B220^+^ IgM^−^ CD43^low^) and pro-B (B220^+^ IgM^−^ CD43^low^) cells derived from the BM of *Fanca*^−/−^ and WT mice (mean ± SEM; n = 5 per genotype).

Because N-nucleotide additions depend on TdT activity, the observed excess of N-additions in the Vκ-Jκ1 rearrangements in *Fanca*^−/−^ mice suggests that the absence of Fanca stimulates TdT action or expression/stabilization. Accordingly, even if Fanca-deficient pro-B and pre-B cells showed similar levels of TdT mRNA (Fig. 4A), pro-B cell population from *Fanca*^−/−^ mice express significantly more protein that their Fanca-proficient littermates, as determined by flow cytometry (Figure S2) and shown in Fig. 4B. Our analysis indicates that Fanca-deficient pre-B cells present a residual level of TdT expression slightly more elevated than in Fanca-proficient cells. Although the differences between the two genotypes is not statistically significant, it could affect N-additions in the Vκ-Jκ1 rearrangements in pre-B cells.

Collectively, these data indicate that, during Vκ-Jκ1 recombination in IgM^−^ B-cells, Fanca loss-of-function specifically results in the accumulation of both in-frame rearrangements and N-nucleotide additions.

### *Fanca* ^−/−^ mice displayed skewed Vκ gene usage in in-frame Vκ-Jκ1 rearrangements in BM IgM^−^ B cells

**Figure 5:**
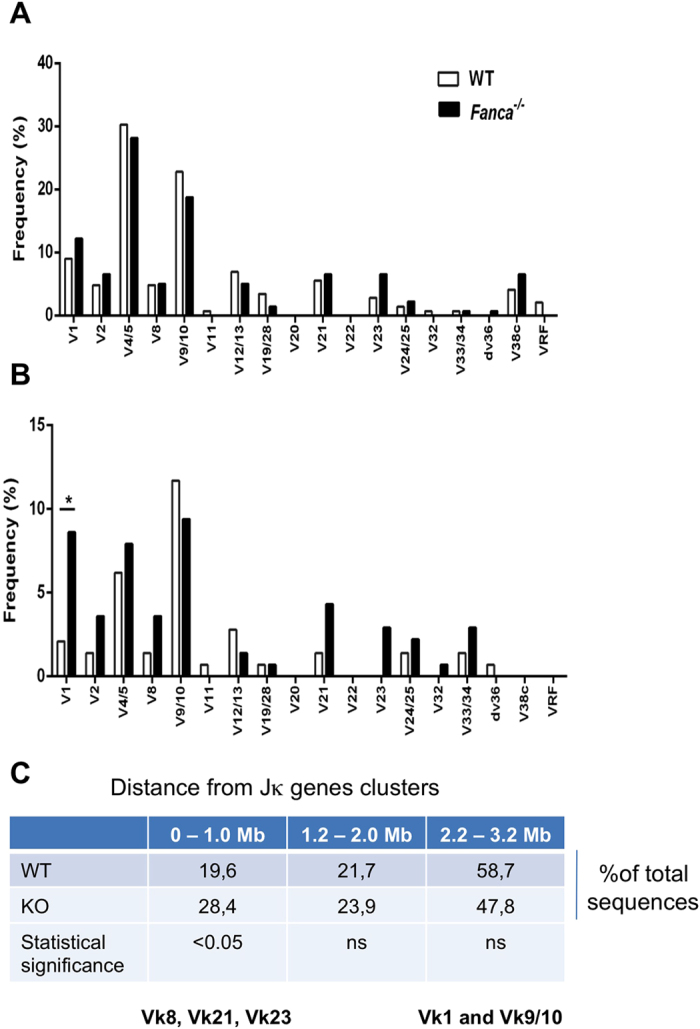
*Fanca*^−/−^ mice displayed a skewed Vκ gene usage in in-frame Vκ-Jκ1 rearrangements in BM IgM- B cells. Analysis of Vκ gene family usage of total (**A**) and in-frame (**B**) Vκ-Jκ1 rearrangements amplified from genomic DNA isolated from BM IgM^−^ B cells of *Fanca*^−/−^ and WT mice (*p < 0.05 with Fisher’s exact test). Vκ families are displayed according to chromosomal order relative to the Jκ genes cluster. The data are from four independent pools of four mice per genotype (numbers of analysed sequences are indicated in Table 1).

Next, we compared the Vκ repertoire usage in Vκ-Jκ1 and Vκ-Jκ4 rearrangements in BM IgM^−^ B cells from *Fanca*^−/−^ and WT mice. Regarding the total Vκ-Jκ1 rearranged junctions, *Fanca*^−/−^ and WT mice shared a similar Vκ family usage profile, with Vκ1, Vκ4/5 and Vκ9/10 used more often (>50%), and the single-members Vκ11, Vκ22, VκRF, and Vκdv36 used rarely, as previously reported^26^,^27^,^28^ (Fig. 5A). Remarkably however, with respect to in-frame Vκ-Jκ1 rearrangements, we noticed the presence of a higher than expected proportion of Vκ1, 2, 8, 21 and 23-family junction in *Fanca*^−/−^ mice. Furthermore, the Vκ1 family was the most significantly increased in *Fanca*^−/−^ mice. Interestingly the Vκ8 Vκ21 Vκ23 gene families are located less than 1.0 Mb from Jκ1, whereas Vκ1 is located more than 2 Mb away (Fig. 5C). A similar analysis of Vκ gene usage in Vκ-Jκ4 rearrangements failed to show differences with respect to both distance and Vκ family usage between WT and *Fanca*^−/−^ mice (Figure S3) further supporting the specificity of the previous observation.

Altogether, our findings indicate that the absence of Fanca specifically leads to altered Vκ gene usage in the in-frame Vκ-Jκ1 rearrangements.

## Discussion

In this study, we used *Fanca*^−/−^ mice to investigate a potential function for the FANC pathway in V(D)J recombination. We found that the absence of Fanca leads two subtle but consistent molecular abnormalities during the process of both HC and LC formation. Whereas the first observed molecular alteration occurred during HC formation with no impact in the pro-B to pre-B transition, the second is associated to a defect in pre-B to immature B-cell transition. However, because of the selection process to which B-cells are subjected to become fully competent, the observed alterations seems have only a modest impact on Ig diversity and functionality.

During the process of HC formation, two rounds of rearrangement follow one to another to allow, first, the junction of a D sequence with a J_H_ sequence and, second, the joining of a V_H_ sequence with the rearranged DJ_H_ sequence. Following the RAG-mediated hairpin formation at CE sequences, rearrangement proceeds thank to the opening of each hairpin, by the joint action of DNA-PK and Artemis^7^, followed by the remodelling of the opened extremities and their joining. The remodelling of the open extremities eventually leads to N and P nucleotide additions at coding joins. P-additions are the consequence of asymmetric opening of hairpin loops that form at gene ends during the HC rearrangement process. The extension of the ss extremities created by the hairpin opening, thus creating a palindrome of 0 to 4 nucleotides at the end. P nucleotides have been associated with V_H_, J_H_ and D genes. Unexpectedly, in this work we noticed that the frequency of P-nucleotides addition during the DJ_H_ rearrangement was significantly more elevated in Fanca^−/−^ than in WT cells, whereas the opposite was observed for the V_H_D rearrangement. Thus, whereas in WT cells the frequency of P-additions is similar at DJ_H_ and V_H_D, in Fanca^−/−^ cells we found 3 times more P-additions at DJ_H_ than at V_H_D junctions. An elevated frequency of sequences with P-additions, but at both DJ_H_ and V_H_D junctions, was previously reported in X- linked anhidrotic ectodermal dysplasia with hyper-IgM syndrome (HED-ID), a rare pathology due to a genetic determined deficiency in NF-kB activation^29^. In HED-ID exacerbated P-nucleotide additions have been directly associated to an altered exonucleolytic processing of the coding ends.

Furthermore, it has been robustly ascertain that cells with a loss-of-function of one FANC protein, in addition to an increase of NF-kB activity^30^,^31^, generally associate an exacerbated use or activity of the NHEJ pathway^20^,^21^,^22^. Indeed, inappropriate recruitment of DNA-PKcs at the site of DSBs was observed in the FANCD2-, FANCC- and FANCA-deficient cell^20^,^22^. Since the number of nucleotides found at the coding ends is the result of the balance between the nucleotide addition and the level of exonuclease activity that occurred before germline ends joining, we hypothesized that the observed differences in P additions is due to alterations in the action of Artemis or the DNA PKcs-Artemis complex during the opening of the hairpin leading to longer ssDNA extremities, a defect not sufficiently compensated by exonucleolytic activities. Indeed, in addition to the endonucleolytic activity of the DNA PKcs-Artemis complex, Artemis alone display an exonucleolytic activity^7^. Although It is not well known when Artemis is free from DNA-PKcs during the process of V(D)J recombination, one can speculate that in the absence of FANCA and/or in the presence of an increase of NF-kB activity, its activity is selectively reduced during DJ_H_ joing and thus leaving more P nucleotides. Nevertheless, we cannot exclude the possibility that V_H_DJ_H_ length selection process may also contribute to the length of P and N nucleotide in both D-J_H_ and V_H_-DJ_H_ ends.

Looking at LC formation, we observed that the rearranged CDR3 show an excess of N-nucleotide additions. (and the recalled parallel deficit in P-nucleotide additions). It is important to note that the structure of the DNA ends created during V(D)J recombination differs markedly from other classical DNA double-strand breaks. Indeed, the V(D)J CEs are protected by a closed hairpin structure. The CE configuration is subject to a concomitant end-processing and sealing process. Furthermore, the end-joining step is accompanied by considerable DNA-end modifications, which contribute to the diversification of the pre-immune repertoire. Following the opening of the hairpin mainly mediated by the endonucleolytic activity of Artemis, the extremities are processed by various polymerases, including the lymphoid-specific enzyme TdT. For B cells, TdT is a key factor in the expansion of the diversity of their repertoire by means of the addition of N-nucleotides onto the ends of the newly formed P-nucleotides. TdT adds an average of two to five nucleotides per N region, and G:C pairs are added more often than A:T pairs^32^. However, the number and sequence of both P- and N-nucleotides that are added vary for each junction. B cells express TdT during the pro-B stage of development, as the heavy chain is produced. N nucleotides are added to both the V-D junctions and the D-J junctions of Ig HC. Once a functional HC is made, the expression of TdT is down-regulated^33^,^34^. The amount of TdT expressed *in vivo* correlates with the degree of N region diversity in the antigen receptor. Finally, it is accepted that N nucleotide addition contributes significantly to the diversity of the HC, whereas few LC rearrangements include the addition of N nucleotides. We observed that, while the levels of TdT mRNA in both pro-B and pre-B cells was similar in WT and *Fanca*^−/−^ mice, the protein level was significantly higher in pro-B cells from *Fanca*^−/−^ mice compared with WT animals (Figs 4B and S2). Noteworthy, although not significant differences are observed in the aggregate data analysis (Fig. 4B), TdT protein level in Fanca deficient pre-B cells is often slightly more important than in their Fanca-proficient counterpart supporting the possibility that the observed increase in nucleotide N-additions in *Fanca*^−/−^ mice at this stage is a consequence of this residual higher expression inherited from the higher expression in the earlier pro-B stage. Interestingly, several published works have demonstrated a requirement of the FANC pathway for either the optimal activity or the stabilization of some TLS polymerases^25^,^35^,^36^, which suggests a broad, direct or indirect, role for the FANC pathway in the turnover and the management of the DNA polymerase functions that are not involved in bulky DNA replication/repair. We propose that Fanca participates by unknown mechanism to the regulation of TdT turnover in mice pro-B cells. In the absence of Fanca, the half-life of TdT increases and, as a consequence, at the pre-B stage the cells still will display an elevated residual amount of the protein potentially responsible for the increase of N addition during the Vκ-Jκ1 rearrangement.

We also observed that the frequency of in-frame Vk-Jk1 sequences retrieved from B cells was more important in *Fanca*^−/−^ mice than in their WT littermates. Additionally, the Vk sequences chosen to join with the Jk1 sequences were generally skewed towards the more proximal Vk sequences (i.e., within less than 1.0 Mb), such as the Vk8, 21 and 23 sequences. The Vk1 sequence is a notable exception: it was more than 2.0 Mb away but was more frequently used (Fig. 5). The first rearrangements at the κLC locus most often employ the Jκ1 segment and the closest Vκ segments^37^. Once an in-frame and productive Vκ-Jκ exon has been successfully created, the B-cells express a κLC that is verified for functionality by pairing with a μHC, thus forming a BCR that becomes an immature (sIgM^+^) B-cell^4^. Our observations suggest that Fanca may be involved in the regulation of the expression and/or pairing of the rearranged LC with a μHC. Indeed, although in-frame, a high proportion of the *Fanca*^−/−^*-*derived small pre-B cells bearing rearranged LC failed to fully progress to the immature B-cell compartment. The higher frequency of in-frame Vκ-proximal-Jκ1 junctions (Fig. 3C) supports the hypothesis that even though they are in-frame, the LCs are not expressed or are unable to pair with a μHC in Fanca-deficient cells. Interestingly, Vk1, which is a distal Vk sequence located more than 2.0 Mb from Jk1, is the most overrepresented between the Vk-Jk1 in-frame retrieved sequences. Interestingly, Vk1 is the only Vk family member that possesses potential NF-κB binding sites in their intronic regions^38^. In the last few years several studies including our own unpublished observations revealed some as yet poorly understood transcriptional activities of the FANC pathway that could both increase or inhibit the transcription of several genes^39^,^40^. Interestingly, it was demonstrated that FANCD2 protein can inhibits NF-κB-dependent transcription through a specific association between monoubiquitinated FANCD2 and a NF-κB consensus-binding site^39^. Moreover, an aberrant activation of NF-κB-dependent transcriptional activity has been observed in FA cells^30^,^31^. Taken together these observations, we speculate that Fanca deficiecy in pre-B cells leads to enhanced NF-κB mediated transcription activity which in turn deregulate the Vk1 expression and managing. A constitutive expression of this particular rearrangements VJ exons can explain both an accumulation of in frame Vκ-Jκ1 rearrangement and an increase load of N addition at this specific rearrangement. In an alternative but not exclusive manner, the observed abnormality could be associated to the higher than normal NHEJ activity associated to the loss-of-function of the FANC pathway. In particular, we have reported that in absence of FANCcore complex or FANCD2 proteins, 53BP1 accumulate strongly and stay longer than in WT cells to DSBs^22^. Thus, an altered 53BP1 accumulation during the Vk-Jk recombination process could greatly favour the joining of proximal DSB, as observed here. Alternatively, 53BP1 and FANCA could be indirectly involved in this DNA end joining process by facilitating chromosomal accessibility or influencing chromatin organization. Although the exact role of 53BP1 in the absence of FANCA in DNA DSB repair remains to be determined, the two proteins appear to function in coordinating certain aspects of DNA end joining during the light chain rearrangement.

Recently we have shown that the Fanca (and likely the FANC pathway) plays a role, alone or in cooperation with other factors, during the antigen-dependent diversification phase of the Ig genes^25^. In this work we showed that Fanca not only plays a role in the nucleotide addition at the CE via the regulation of TdT protein expression/stabilization but also point out to the role in the expression of the LC Vk1 family. Nevertheless, because of the huge repertoire of V, (D), and J segments and the selection process to which B cells are subjected to become fully competent, the observed alterations have, at least in mice, only a modest impact on Ig diversity and functionality.

## Materials and Methods

### Mice

*Fanca*^−/−^ mice were described previously^25^ *Fanca*^*+/−*^ mice were backcrossed with WT FVB/N mice (>ten generations). As *Fanca*^−/−^ mice show severely reduced fertility, WT and *Fanca*^−/−^ mice used for analysis correspond to siblings derived from crossbreeding of heterozygous mice. The project was officially approved by the Animal Experimentation Ethics Committee of the Gustave Roussy Institute (IGR) and registered under no. 26 by the IGR Department of Research and conducted in accordance with French laws and regulations.

### Flow cytometry

BM was harvested by flushing tibias and femurs. Freshly isolated bone marrow or splenocytes were filtered and immunostained for 20 minutes at 4 °C in PBS–0.5% bovine serum albumin, with fluorochromes conjugated antibodies. Single-cell suspensions of the BM and the spleen were analysed using BD Accuri™ C6 system (BD Biosciences, San Jose, CA, USA) after staining with the following antibodies: anti-IgM-FITC (eB121-15F9, eBioscience, San Diego, CA, USA); anti-B220-PE (RA3-6B2, BioLegend, San Diego, CA, USA); anti-CD43-biotin (S7, BD Pharmingen, San Jose, CA, USA); and streptavidin-APC (eBioscience, San Diego, CA, USA). Control samples included unstained cells, single-color controls, and “fluorescence minus one” controls. Doublets were excluded by plotting SSC-A versus SSC-W. Sorting were performed using BD-Influx Cell Sorter with the purity of sorting >95%. BM B220^+^ IgM^−^ cells were sorted using a Moflo cell sorter (Cytomation). Gating strategies are presented in Figure S1 and S2.

### PCR assay for κLC rearrangement

Genomic DNA was isolated from sorted BM B220^+^ IgM^−^ cells. DNA was analysed by PCR with different cycles and by Southern blots for Vκ-Jκ rearrangement as previously described^41^.

### Sequence analysis of Ig gene rearrangements

Genomic DNA was extracted from sorted BM B220^+^ IgM^−^ cells. V_H_DJ_H4_, Vκ-Jκ1 and Vκ-Jκ4 rearrangements were amplified by PCR as previously described^42^. PCR products were subsequently cloned into the Zero Blunt vector (Invitrogen) and sequenced. All V(D)J recombined products were analysed with the IgBLAST webserver (NCBI).

### Flow cytometry for TdT staining

Cells were stained for surface antigens using anti-B220-PacificBlue, anti-IgM-FITC, anti-CD43-APC (all from eBioscience, San Diego, CA). Then, a fixation/permeabilization procedure was performed using the Foxp3 Staining Buffer Set (eBioscience) according to the manufacturer’s protocol, followed by staining with anti-TdT-PE (19−3; eBioscience). Cells were analysed with an LSR II flow cytometer with FlowJo (TreeStar Inc., Ashland, OR, USA) software.

### qRT-PCR

The total RNA from sorted pro-B and pre-B cells was isolated using the RNeasy Plus Micro kit (Qiagen, Hilden, Germany) and reverse-transcribed using an Affinity Script Multi Temperature cDNA synthesis kit (Agilent Technologies, Santa Clara, CA, USA). All reactions were performed using the Fast Start Universal SYBR Green Master mix (Roche, Penzberg, Germany) and a 7300 Real-Time PCR System (Applied Biosystems, Foster City, CA, USA) with the following primers: TdT: Forward, AGAGACCTTCGGCGCTATG; Reverse, TGACAGTCTTCCCCTTAGTCC; Β actin: Forward, GACGGCCAGGTCATCACTATTG; Reverse, AGGAAGGCTGGAAAAGAGCC.

## Additional Information

**How to cite this article**: Nguyen, T. V. *et al*. V(D)J recombination process and the Pre-B to immature B-cells transition are altered in *Fanca*^*−/−*^ mice. *Sci. Rep.* **6**, 36906; doi: 10.1038/srep36906 (2016).

**Publisher's note:** Springer Nature remains neutral with regard to jurisdictional claims in published maps and institutional affiliations.

## Supplementary Material

Supplementary Information

## Figures and Tables

**Table 1:** CDR3 lenght of heavy and light chain rearrangements inFanca^−/−^ and WT mice.

Ig gene rearrangement^a^	Mice	Average CD3 lenght in bp (number of analyzed sequences)	CDR3 size range in bp	% in-frame sequences	% out-of-frame sequences	In-frame *vs* out-of -frame ratio
VDJ_H4_	WT	40.5 (54)	30–61	78	22	3.54
	*Fanca*^−/−^	39.4 (42)	24–57	83	17	4.88
Vk-Jk1	WT	26.4 (145)	15–34	32	68	0.47
	*Fanca*^−/−^	26.4 (139)	18–32	48	52	0.92
Vk-Jk4	WT	26.1 (93)	17–31	30	70	0.42
	*Fanca*^−/−^	26.3 (82)	17–33	28	72	0.38

^a^Rearrangements were amplified from genomic DNA isolated from BM B220^+^ IgM^−^ cells.
