# Supplementary material for: V(D)J recombination process and the Pre-B to immature B-cells transition are altered in Fanca−/− mice
Source: Sci Rep. 2016 Nov 24;6:36906. doi: 10.1038/srep36906 (PMC5121645; doi:10.1038/srep36906)
Supplement: Supplementary Information [file srep36906-s1.pdf]

SUPPLEMENTAL INFORMATIONS

V(D)J recombination process and the Pre-B to immature B-cells transition are altered in *Fanca*$^{-/-}$ mice.

Thuy Vy Nguyen, Patrycja Pawlikowska Virginie, Firlej, Filippo Rosselli and Saïd Aoufouchi

Table S1. Proportions of different B cell compartments in BM

Table S2. Summary of Vk-Jk1 junctions in the different experiments realized. The data were utilized to draw figure 2B and 2C.

Figure S1. Gating strategy used in TdT protein analysis by flow cytometry. The TdT positive cells in pre-B (B220^{+} IgM^{-} CD43^{low}) and pro-B (B220^{+} IgM^{-} CD43 CD25^{low}) compartments derived from BM of *Fanca*$^{-/-}$ and WT mice.

Figure S2. Gating strategy used for sorting pro-B and pre-b cells from BM of *Fanca*$^{-/-}$ and WT mice.

Figure S3. Fanca-deficient mice showed normal Vκ gene usage in Vκ-Jκ4 rearrangements in BM IgM^{-} B cells. Analysis of Vκ gene family usage of total (A) and in-frame (B) Vκ-Jκ4 rearrangements amplified from genomic DNA isolated from BM IgM^{-} B cells of *Fanca*$^{-/-}$ and WT mice. Vκ families are displayed according to chromosomal order relative to the Jκ genes cluster. Data are from three independent pools of three mice per genotype (numbers of analyzed sequences are indicated in Table 1).

Figure S4. Original images used for figure 3B. Dotted lines enclose the images reported in Figure 3. In A, three different exposures of the same gel on the same film were showed. In B, the scan of the original PCR gel was presented, before the inversion of the black and white colors.

Table S1. Proportions of different B cell compartments in BM

	IgM^-		IgM^+ $B220^{low}$		IgM^+ $B220^{high}$	
	WT	$Fanca^{-/-}$	WT	$Fanca^{-/-}$	WT	$Fanca^{-/-}$
Mean (n=8)	15.2	15.9	5.3	4.6	8.7	8.5

Supplemental Table 1 – Nguyen et al.

Table S2. Summary of Vκ-Jκ1 junctions

	In-frame (no. seq)		N-addition (no. seq)		P-addition (no. seq)		Total no. seq	
	WT	*Fanca-/-*	WT	*Fanca-/-*	WT	*Fanca-/-*	WT	*Fanca-/-*
Exp1	11	16	2	2	3	1	38	39
Exp2	11	17	0	3	6	2	30	32
Exp3	10	20	1	6	2	3	39	43
Exp4	15	14	1	1	3	0	38	25
Total	47	67	4	12	14	6	145	139

Supplemental Table 2 – Nguyen et al.

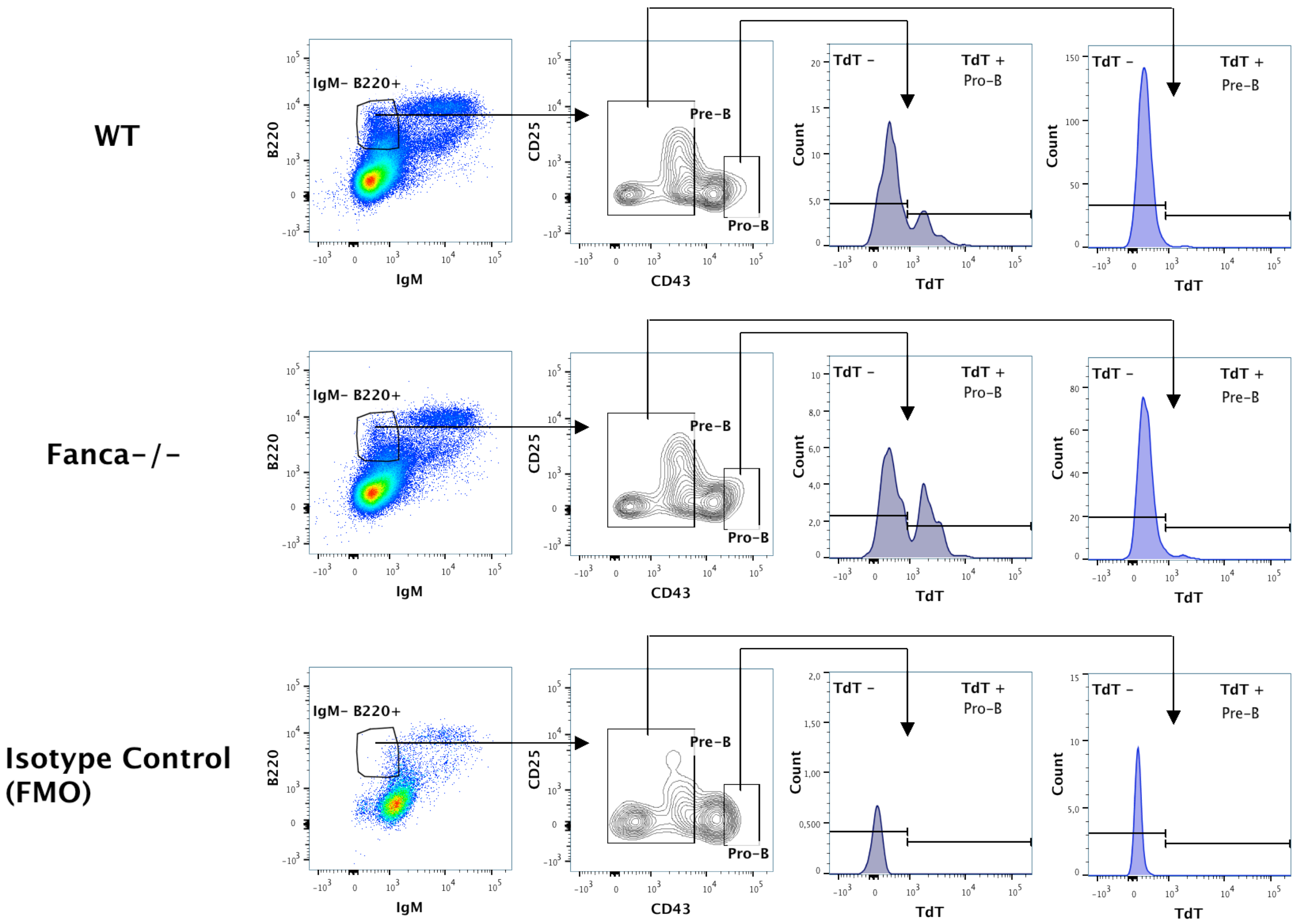

Supplemental Figure 1 – Nguyen et al.

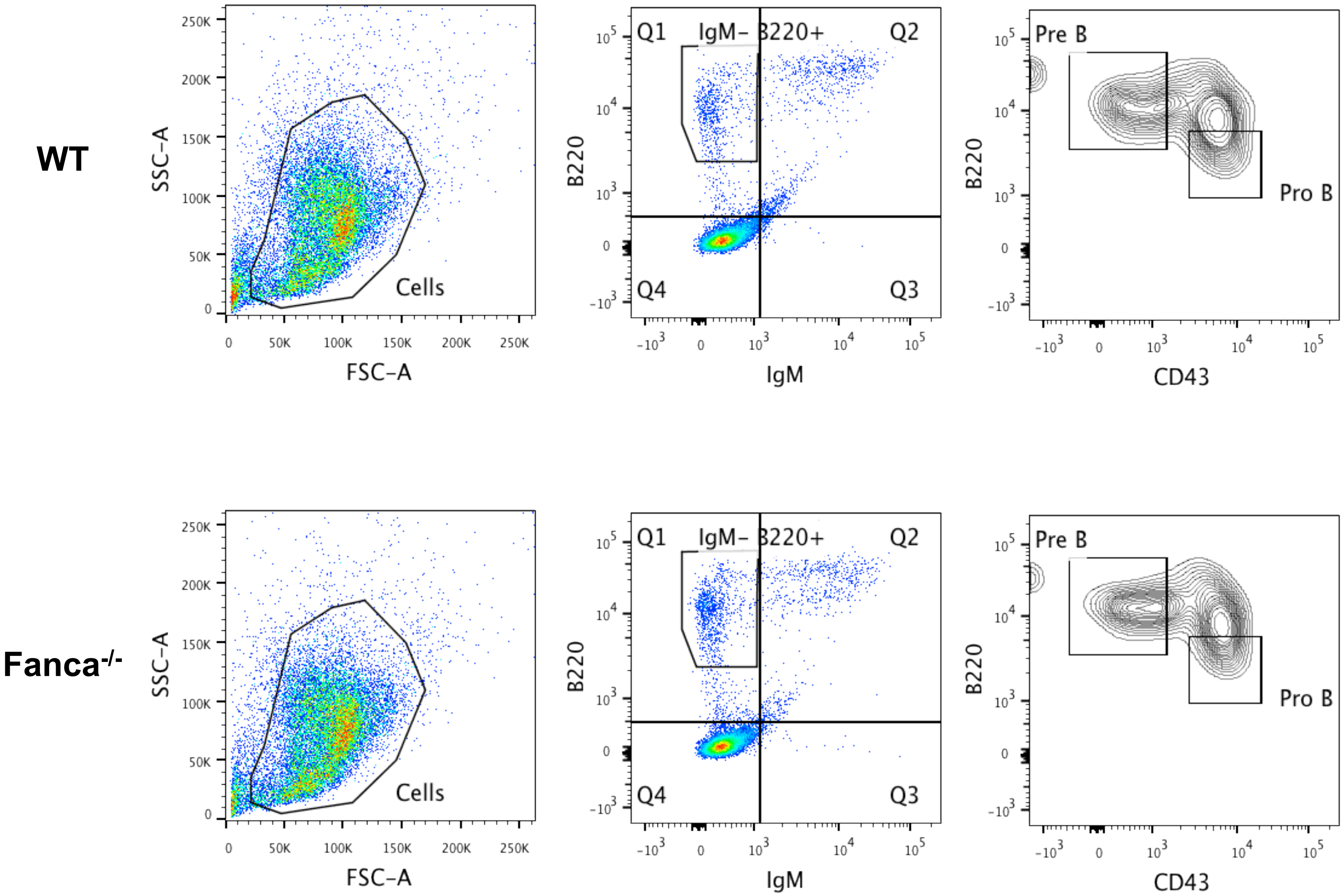

Supplemental Figure 2 – Nguyen et al.

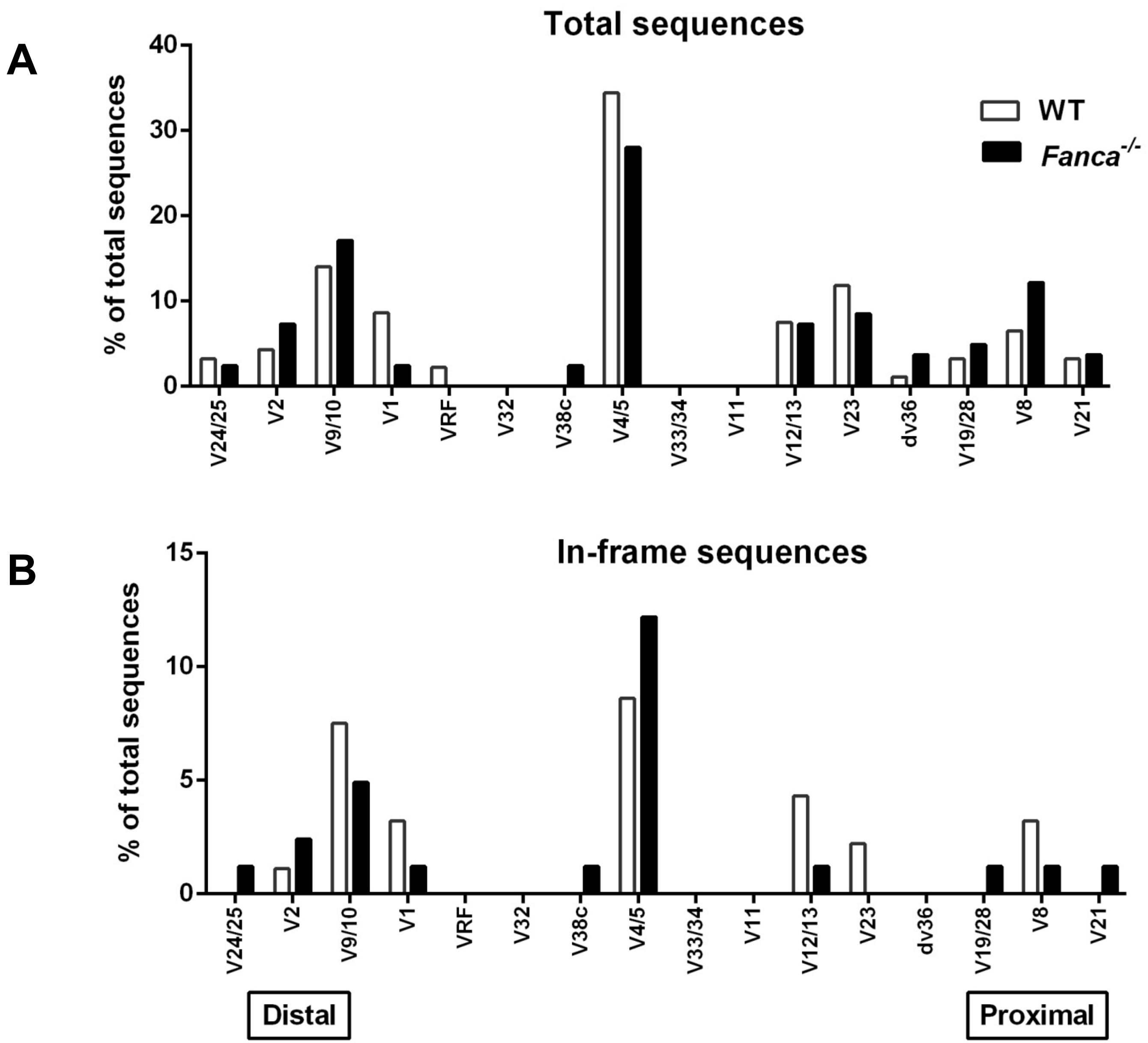

Supplemental Figure 3 – Nguyen et al.

A

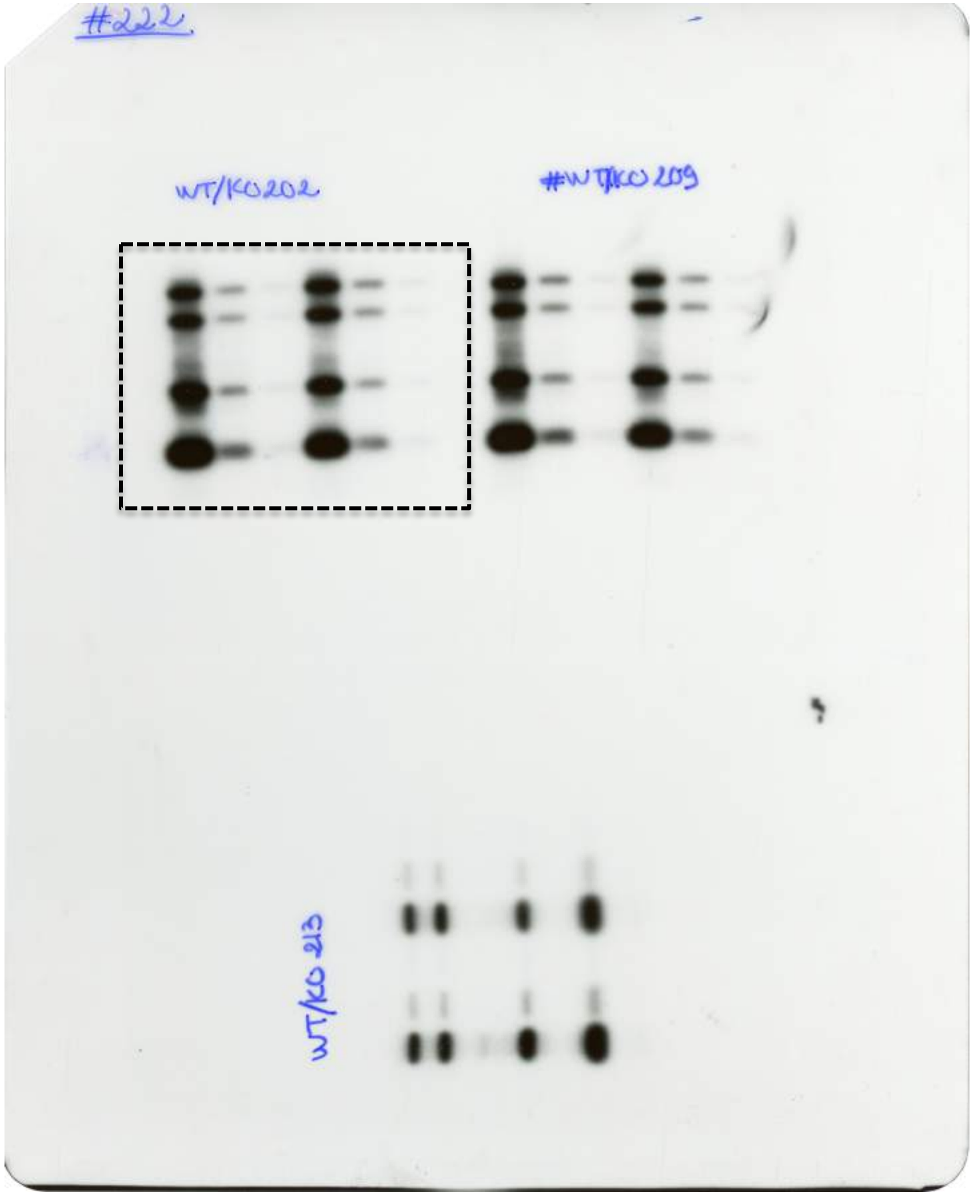

B

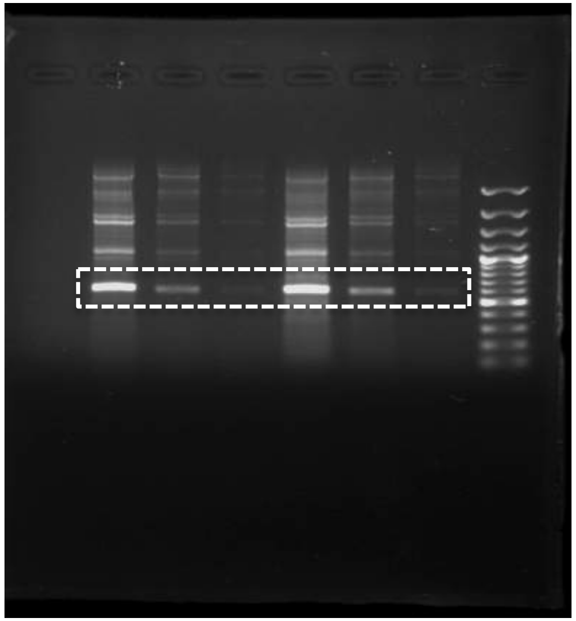

Supplemental Figure 4 – Nguyen et al.
